# Dynamic Analysis of the Locomotion Mechanism in Foxtail Robots: Frictional Anisotropy and Bristle Diversity

**DOI:** 10.3390/biomimetics9060311

**Published:** 2024-05-22

**Authors:** Jaegak Lee, Hyemi Jeong, Taehyun Kim, Hyunseok Yang

**Affiliations:** Department of Mechanical Engineering, Yonsei University, 50, Yonsei-ro, Seodaemun-gu, Seoul 03722, Republic of Korea; jaegak@yonsei.ac.kr (J.L.); stree104@yonsei.ac.kr (H.J.); fireboarder81@gmail.com (T.K.)

**Keywords:** biomimatic robot, frictional anisotropy, soft robotics, bristle robot

## Abstract

This study investigated the locomotion mechanism of foxtail robots, focusing on the frictional anisotropy of tilted bristles under the same friction coefficient and propulsion strategy using bristle diversity. Through dynamic analysis and simulations, we confirmed the frictional anisotropy of tilted bristles and elucidated the role of bristle diversity in generating propulsive force. The interaction between contact nonuniformity and frictional anisotropy was identified as the core principle enabling foxtail locomotion. Simulations of foxtail robots with multiple bristles demonstrated that variations in bristle length, angle, and deformation contribute to propulsive force generation and environmental adaptability. In addition, this study analyzed the influence of major design parameters on frictional anisotropy, highlighting the critical roles of body height, bristle length, stiffness, reference angle, and friction coefficient. The proposed guidelines for designing foxtail robots emphasize securing bristle nonuniformity and inducing contact nonuniformity. The simulation framework presented enables the quantitative prediction and optimization of foxtail robot performance. This research provides valuable insights into foxtail robot locomotion and lays a foundation for the development of efficient and adaptive next-generation robots for diverse environments.

## 1. Introduction

**Figure 1:**
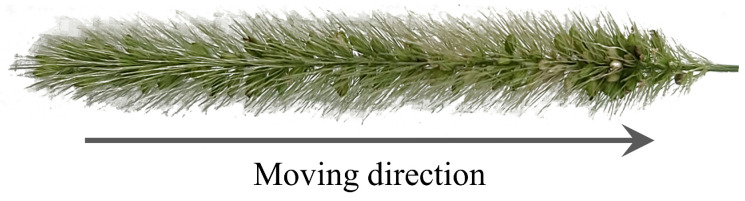
Photograph of a foxtail millet inflorescence. When the foxtail is grasped and released repeatedly, it moves in the direction opposite to the tilted bristles, as indicated by the arrow.

Biomimetic robots, designed to mimic the form, movement, or behavior of living organisms, have garnered significant interest because of their potential for efficient and adaptive locomotion in complex environments. Foxtail robots, inspired by the distinctive structural and kinematic characteristics of foxtail millet inflorescence (Figure 1), have demonstrated the potential to navigate confined spaces such as pipes or narrow passages, where conventional wheeled or tracked robots encounter challenges [1].

**Figure 2:**
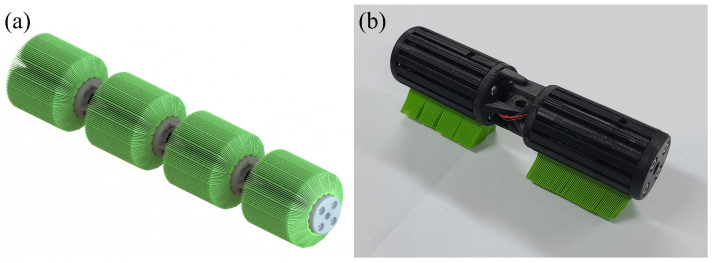
The foxtail robot. (**a**) CAD model of the foxtail robot, showcasing its main body, tilted bristle arrays, and the carangiform locomotion mechanism. (**b**) The foxtail robot under construction using 3D-printed PLA parts, demonstrating the implementation of the proposed design.

**Figure 3:**
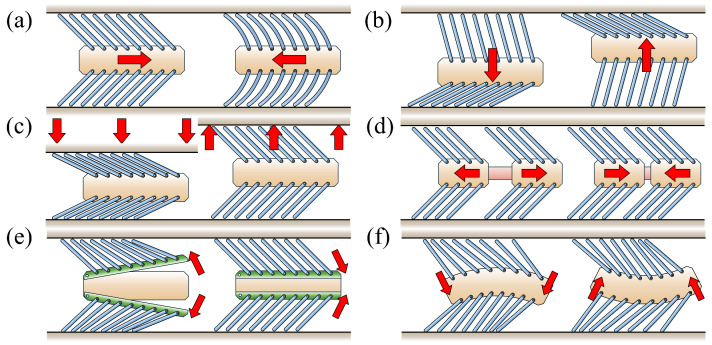
Six types of foxtail robot locomotion mechanisms. The red arrows indicate the direction of the forces: (**a**) horizontal external force; (**b**) vertical external force; (**c**) pipe height change; (**d**) inchworm mechanism; (**e**) phase-difference mechanism; (**f**) carangiform locomotion.

The locomotion of foxtail robots relies on the tilted bristle array structure (Figure 2), which facilitates easy sliding in the direction opposite to the bristle tilt while resisting motion toward the tilt. This phenomenon, known as frictional anisotropy, is the key principle that enables the propulsion of foxtail robots. Various operation methods for foxtail robots have been proposed, including the use of external forces (Figure 3a–c) such as horizontal external forces [2,3], vertical external forces [4,5,6], and pipe height changes [7]. Internal actuation mechanisms (Figure 3d–f) such as the inchworm [8,9], phase-difference [10], and carangiform locomotion [1] have also been studied. Detailed discussions on the simulations and analyses of these diverse operational methods are presented in Section 4.

Previous studies have demonstrated the operation of foxtail robots using various mechanisms. However, these studies lacked an in-depth analysis of the locomotion principles underlying the robots’ functionality. Recognizing this limitation, the present research aimed to conduct a theoretical and systematic analysis of the locomotion mechanism of foxtail robots.

Frictional anisotropy is also used as a major locomotion principle in vibration robots, a type of bristle robot [11,12,13,14,15,16]. These robots operate by applying high-frequency vibrations, ranging from a minimum of 10 Hz [11] to several tens of kHz [16], in either the vertical or horizontal direction. When applied to angled bristles exhibiting frictional anisotropy, these vibrations propel the robot, with the direction of motion being dependent on the vibration frequency.

However, the locomotion mechanism of foxtail robots differs significantly from that of vibration robots. While foxtail robots achieve movement through large, quasi-static deformations of bristles at lower frequencies, vibration robots rely on high-frequency vibrations. Consequently, directly applying existing dynamic models of vibration robots to foxtail robots poses challenges. To gain a precise understanding of foxtail robot locomotion, a novel dynamic model that accounts for the substantial deformation of bristles is needed.

Despite numerous studies on foxtail robots, existing research has attributed the frictional anisotropy to variations in friction coefficients, which is counterintuitive under the same material conditions [7,10]. Moreover, the impact of bristle diversity on a robot’s propulsion strategy remains insufficiently explored. In the field of biology, a study analyzed the frictional anisotropy of insect setae using a cantilever beam model [17]. Setae are hair-like structures on the legs of insects that serve a function similar to that of the bristles in foxtail robots. However, this study also attributed anisotropy to differences in friction coefficients.

To address these limitations, this study aimed to develop a comprehensive dynamic model that precisely characterizes the locomotion principles of foxtail robots. Employing multi-body dynamic analysis and simulations, we elucidate the mechanism through which frictional anisotropy manifests under the same friction coefficient. In addition, our investigation aimed to understand the fundamental locomotion principles of the robot. By identifying key design factors and assessing their impact on locomotion performance, we provide practical guidelines for optimizing the design of foxtail robots.

The insights gained from this study, particularly regarding the role of bristle diversity, are expected to advance our understanding of foxtail robot locomotion principles. These findings can guide the development of efficient and adaptive next-generation robotic systems for diverse, challenging environments. Furthermore, the proposed simulation framework enables the quantitative prediction and optimization of foxtail robot performance. The remainder of this paper is organized as follows. Section 2 details the methodology, including the ground contact model, linkage-spring bristle model, a dynamic analysis using the Euler–Lagrange method, and specifications. Section 3 presents the analysis of frictional anisotropy in a tilted bristle and the simulation results of the bristle model. Section 4 discusses the simulation results of the foxtail robot model under various scenarios. Section 5 provides a discussion on the implications of the findings, their potential applications, and the limitations of this study. Finally, Section 6 concludes the paper and outlines future research directions.

## 2. Methodology

This section presents the modeling techniques and simulation methodology used to analyze the locomotion mechanism of foxtail robots. We explain three key components: the ground contact model, which simulates the frictional anisotropy of bristles (the core principle of foxtail robots); the linkage-spring model, which implements the elastic deformation of bristles; and the Euler–Lagrange method, which is used to analyze the overall system dynamics. In addition, the specifications of the simulated foxtail robot, including its dimensions and physical properties, are presented.

### 2.1. Ground Contact Model

**Figure 4:**
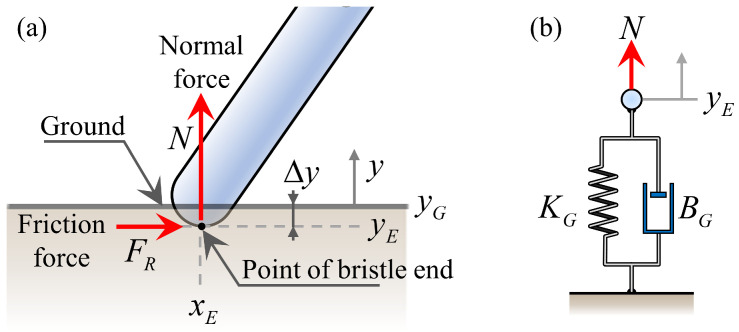
Ground contact: (**a**) schematic of the bristle-ground contact, showing the penetration of the bristle end into the ground. The height of the bristle end and the ground are denoted by $y_{E}$ and $y_{G}$, respectively. (**b**) The bristle–ground interaction is modeled as a spring-damper system. The normal force *N* is calculated based on the linear Kelvin–Voigt contact model.

To accurately model the frictional anisotropy of the bristles, we must calculate the normal force generated by the contact between the bristles and the ground. Our approach considers the interaction between the ground and the bristles as a spring-damper system. Specifically, we adopted the linear Kelvin–Voigt contact model [18], which calculates the normal force proportional to the penetration depth. As shown in Figure 4a, when the height of the bristle end $y_{E}$ becomes lower than the ground height $y_{G}$, resulting in a penetration of $\Delta y=y_{G}-y_{E}$, the normal force *N* is calculated through the spring-damper system in Figure 4b. This calculation involves the ground stiffness $K_{G}$ and damping coefficient $B_{G}$, as expressed in Equation (1):

(1) $$N=\left\{\begin{array}{cc} K_{G}\left(y_{G}-y_{E}\right)+B_{G}\left(-{\dot{y}}_{E}\right) & \mathrm{for}\;y_{E}\leq y_{G}\\ 0 & \mathrm{otherwise} \end{array}\right.$$

The frictional force $F_{R}$ exhibits dependence on the object’s motion state, with static friction acting when the object is stationary and kinetic friction acting when it is in motion [19]. This study focused on the frictional anisotropy that occurs under constant friction coefficients, rather than the frictional anisotropy caused by the difference between the maximum static and kinetic friction coefficients. Therefore, the simulation set the maximum static and kinetic friction coefficients to identical values for Equation (2). For velocities lower than the characteristic velocity γ, the static friction coefficient was used.

(2) $$F_{R}=\left\{\begin{array}{cc} -\mathrm{sgn}\left(v\right)\mu_{k}\frac{|{\dot{x}}_{E}|}{\gamma }N & \mathrm{for}\;|{\dot{x}}_{E}|\leq \gamma \\ -\mathrm{sgn}\left(v\right)\mu_{k}N & \mathrm{otherwise} \end{array}\right.$$

### 2.2. Linkage-Spring Model

In this study, we used the linkage-spring model to represent the bristles, which discretized them into multiple links and torsional springs. This enabled a more straightforward dynamic analysis, using the Euler–Lagrange method, than the cantilever beam model. The stiffness of the springs was calculated such that the deformation of the linkage-spring model matched that of the cantilever beam under identical loading conditions. This approach was inspired by a previous study [20] that used a cantilever beam as a linkage-spring model for design.

**Figure 5:**
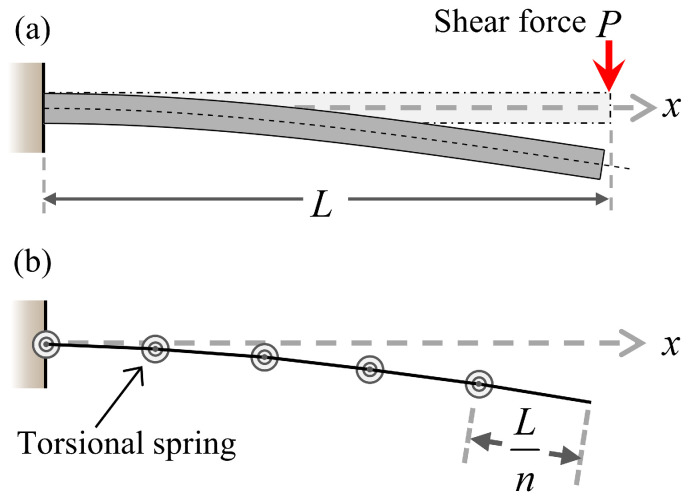
Comparison of the deformation: (**a**) cantilever beam under shear force *P*; (**b**) linkage-spring system configured with *n* links and torsional springs under same shear force.

As shown in Figure 5, the bristles were modeled as a linkage-spring system consisting of *n* links and torsional springs between the links. The stiffness of the first spring, denoted as $k_{1}$, and the stiffness of the remaining springs, denoted as $k_{Else}$, were determined using Equations (3) and (4), respectively. For a comprehensive understanding of this calculation process, please refer to Appendix A.

(3) $$k_{1}=\frac{6n^{2}EI}{\left(3n-1\right)L}$$

(4) $$k_{Else}=\frac{nEI}{L}$$

**Figure 6:**
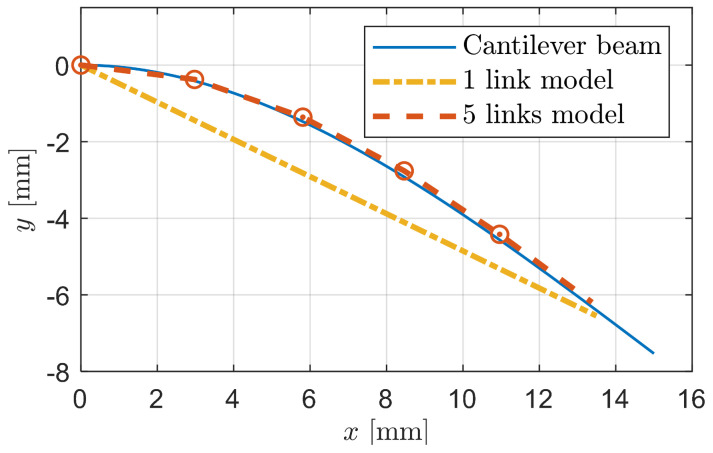
Deformation comparison of a cantilever beam and linkage-spring models with different numbers of links under the same shear force.

Figure 6 illustrates the deformation of the linkage-spring model under the influence of the derived stiffness when subjected to a shear force. For comparison, we also present the deformation of a cantilever beam. The results reveal that the linkage-spring model closely mirrors the deformation pattern of the cantilever beam, with the resemblance becoming more pronounced as the number of links increases.

### 2.3. Dynamic Analysis Using Euler–Lagrange Method

**Figure 7:**
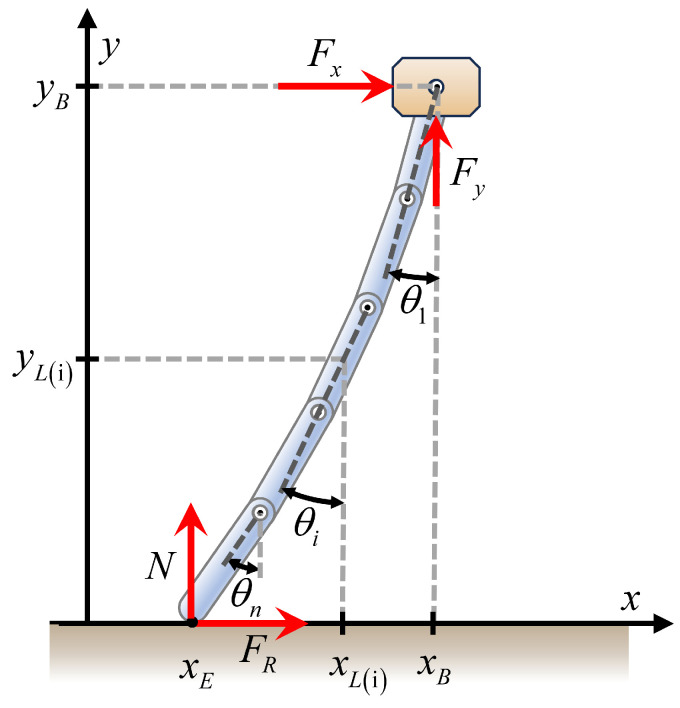
Schematic of the bristle model, showing the body position $\left(x_{B},y_{B}\right)$, the center of mass position of the *i*-th link $\left(x_{L(i)},y_{L(i)}\right)$, the bristle end position $x_{E}$, the rotation angle of the *i*-th link $\theta_{i}$, and the forces acting on the bristle end (normal force *N* and friction force $F_{R}$) and the body (horizontal external force $F_{x}$ and vertical external force $F_{y}$).

The bristles of the foxtail robots have a complex structure comprising multiple links, as shown in Figure 7. When analyzing the dynamics of this multi-body system, employing the Euler–Lagrange method is more efficient than relying on Newton’s laws of motion. This efficiency becomes particularly pronounced when extending from a single bristle model to a foxtail robot model that incorporates multiple bristles.

The position of the body ($x_{B}$, $y_{B}$) and the rotation angles of each link $\theta_{i}$ were denoted as *q*. These variables were collectively represented as the vector q. Subsequently, we calculated the Euler–Lagrange equation for each variable, as expressed in Equation (5), where $Q_{i}$ represents the generalized force term corresponding to each variable $q_{i}$:

(5) $$\frac{d}{dt}\left(\frac{\partial T}{\partial \dot{q}i}\right)-\frac{\partial T}{\partial qi}+\frac{\partial R}{\partial \dot{q}i}+\frac{\partial U}{\partial qi}=Q_{i}$$

In arranging the equations into matrix form, an explicit expression for the acceleration of the main variables $\ddot{q}$ can be obtained, as shown in Equation (6). In this context, A represents the coefficient matrix for acceleration, B is a vector that contains nonlinear terms for velocity and variables, and F denotes the generalized force vector.

(6) $$\ddot{q}=A^{-1}\left(B+F\right)=f\left(q,\dot{q},F\right)$$

This ordinary differential equation was solved using the ode function in MatLab, enabling the simulation of the behavior of the foxtail robot system over time for specified initial conditions.

### 2.4. Specification

To perform simulations, reference values for the physical properties and environmental conditions of the foxtail robot are required. In this study, we assumed that the robot was 3D printed using a polylactic acid (PLA) filament, and we set the corresponding parameter values, as shown in Table 1.

The values for the PLA density and Young’s modulus were obtained from a previous study on 3D-printed materials [21]. Ground stiffness and damping values were referenced from a separate study that modeled robot–ground interactions [22]. Meanwhile, the damping ratio ζ of the PLA is known to vary between 0.01 and 0.05 depending on the output conditions [23,24]. For this simulation, a mid-range value of 0.03 was chosen as the damping ratio.

## 3. Bristle Model

To understand the locomotion mechanism of foxtail robots, it is necessary to thoroughly analyze the dynamic movement of individual bristles. In this section, a bristle analysis was conducted while excluding the influence of the robot body. To achieve this, we assumed a fixed body mass and posture, focusing on scenarios with limited bristle height, which is relevant for the main application environment for foxtail robots: navigating pipes.

The simulation results derived in this section elucidate the influence of the anisotropy and deformation characteristics of bristle friction on robot movement. Furthermore, these results will serve as the basis for the dynamic analysis of the entire foxtail robot system presented in Section 4.

### 3.1. Analysis of Frictional Anisotropy Based on Changes in Major Design Parameters

In this section, we systematically examine the influence of the key design parameters of bristles on frictional anisotropy using the reference specifications outlined in Table 1. The parameters under consideration include the number of links constituting the bristles, body height, bristle length, reference angle, and friction coefficient with the ground.

The evaluation was conducted at a height corresponding to a vertical external force $F_{y}$ of −0.001 N across all cases except for the body height variation experiment. In addition, except for the analysis of the influence of the number of links, the bristles consisted of 10 links.

In each evaluation case, we computed the normal force during the forward and backward bristle movements, assuming that the bristles had achieved a static equilibrium state. To quantitatively evaluate the degree of frictional anisotropy, we used the force ratio (FR), defined as the normal force during backward motion divided by the normal force during forward motion, expressed as $N_{\mathrm{Backward}}/N_{\mathrm{Forward}}$.

**Figure 8:**
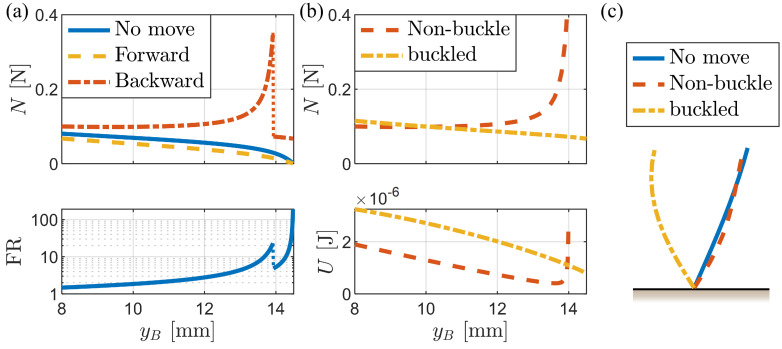
Analysis of frictional anisotropy according to changes in the fixed height of the body ($y_{B}$). (**a**) Graphs showing the normal force and force ratio (FR) as functions of body height. (**b**) Graphs comparing the normal force and potential energy for the buckled and non-buckled states at different body heights. (**c**) Illustrations of the bristle deformation under different conditions. The bristle deformation is shown for the same fixed body height ($y_{B}=13.99$ mm) in each case.

Figure 8a illustrates the variations in the normal force and FR with fixed body height. The y-axis of the FR graph is presented on a logarithmic scale for enhanced visualization. A notable observation is the abrupt change in normal force near the critical height, which is attributed to the buckling phenomenon of the bristles during backward motion (Figure 8c). In addition, the height–potential energy graph (Figure 8b) reveals significant changes in the stable bristle shape before and after reaching the critical height.

For a single-link bristle, the critical height can be calculated by simultaneously solving the static equilibrium equations (Equations (7) and (8)), yielding $y_{B}=l_{L}\cos\left(\tan^{-1}\mu_{k}\right)$, where $l_{L}$ is the link length, *L* is the total bristle length (equal to $l_{L}$ for a single-link bristle), and $\mu_{k}$ is the friction coefficient. Above this critical height, there is no solution for the non-buckling case. In the case of multi-link bristles, the critical height occurs at a slightly higher value but remains close to the approximation $y_{B}=L\cos\left(\tan^{-1}\mu_{k}\right)$.

(7) $$y_{B}=l_{L}\cos\theta_{1}\;(\mathrm{for}\;\mathrm{single-link}\;\mathrm{bristle})$$

(8) $$\left(\sin\theta_{1}-\mu_{k}\cos\theta_{1}\right)Nl_{L}=\frac{1}{2}mLgl_{L}\sin\theta_{1}-k_{1}\left(\theta_{0}-\theta_{1}\right)$$

The bristle deformation is shown for three scenarios: no movement (under no horizontal external force), forward movement, and backward movement. In the backward movement case, the bristles assume a buckled shape, which leads to a discontinuous change in the normal force and high FR, as observed in Figure 8a.

**Figure 9:**
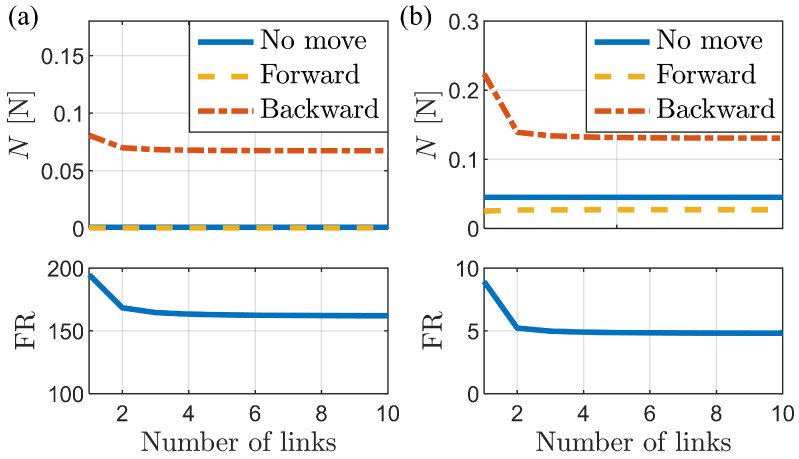
Changes in normal force and frictional anisotropy according to the number of links. (**a**) Normal force and FR vs. number of links for the buckling case (vertical external force: −0.001 N). (**b**) Normal force and FR vs. number of links for the non-buckling case (vertical external force: −0.045 N).

Figure 9 shows the variation in normal force and frictional anisotropy as a function of the number of links in the bristles. The FR remains relatively constant for bristles with five or more links, indicating that the number of links has a limited influence on the frictional anisotropy, except for very low link numbers. The higher FR for the single-link bristle can be attributed to its inability to capture the elasticity and bending behavior of real bristles. A single link prevents bending, thereby eliminating the ability to store elastic energy in the torsional springs between links.

With an increasing number of links, the bristle more accurately reflects the deformation patterns of real bristles. Consequently, both the normal force and FR converge toward constant values. These findings indicate that using sufficient links (e.g., five or more) in foxtail robot simulations is crucial for capturing realistic behavior.

**Figure 10:**
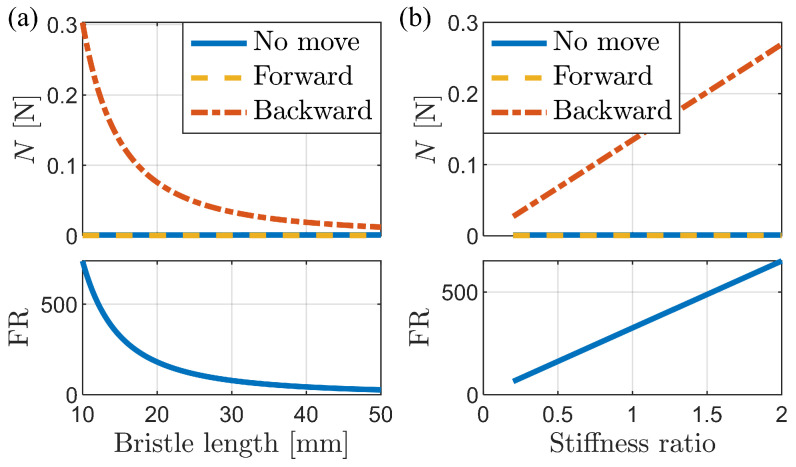
Change in normal force and frictional anisotropy according to (**a**) bristle length and (**b**) stiffness ratio.

Figure 10 shows the variation in frictional anisotropy with respect to changes in bristle length and stiffness ratio. This stiffness ratio is a value multiplied by the stiffness calculated based on the conditions in Table 1 and is used to determine the new stiffness. The observed increase in the FR with decreasing bristle length or an increasing stiffness ratio aligns with the elastic calculation formulas (Equations (3) and (4)). These equations demonstrate an inverse proportional relationship between bristle stiffness and length.

Variation in the stiffness ratio can also occur due to changes in material. Although PLA was used in this study, using different materials can lead to different stiffness ratios. This is because when the material changes, Young’s modulus value changes, resulting in either an increase or decrease in stiffness.

For example, if materials with lower Young’s moduli than that of PLA are used, such as hard ABS (Young’s modulus: 1177 MPa) or soft TPU (Young’s modulus: 2174 MPa), the stiffness of the bristles will decrease, potentially leading to a reduction in frictional anisotropy.

On the other hand, in the case of real foxtail millet plants, although direct data on the Young’s modulus of the bristles could not be found, we instead found the Young’s modulus of the stem, which is approximately 8460 MPa [25]. In this case, as the Young’s modulus is higher than that of PLA, the stiffness of the bristles would increase, potentially leading to an increase in frictional anisotropy.

**Figure 11:**
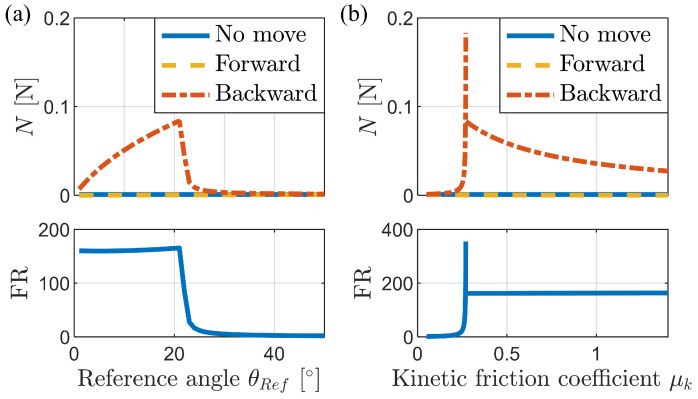
Change in normal force and frictional anisotropy according to (**a**) reference angle and (**b**) kinetic friction coefficient.

Figure 11a illustrates the variations in the normal force and FR with respect to the reference angle. Notably, a significant change occurred around 22°, which can be attributed to the presence or absence of buckling. According to Equation (8), the critical angle for buckling is approximately $\tan^{-1}\left(\mu_{k}\right)$. For $\mu_{k}=0.4$, this critical angle is approximately 21.8°. When the reference angle is below this critical angle, buckling occurs. Conversely, above the critical angle, buckling does not occur. From the perspective of normal force, the backward case exhibits an increasing trend with the reference angle until buckling occurs. However, as the reference angle becomes excessively large (i.e., when the bristles are highly inclined), the frictional anisotropy diminishes. Therefore, it is recommended to determine the reference angle by considering the critical buckling angle, which is calculated based on the friction coefficient.

Figure 11b illustrates the variations in the normal force and FR with respect to the kinetic friction coefficient. Notably, a significant change occurs around the critical friction coefficient $\mu_{k}=\tan\left(15^{\circ }\right)=0.2679$ for a reference angle of 15°. Buckling occurs when the friction coefficient exceeds this critical value, resulting in a high and constant FR. In the non-buckling region, the frictional anisotropy is low, but sharply increases near the critical value. These findings highlight the strong correlation between the friction coefficient and the frictional anisotropy of the bristles, with a sufficiently large friction coefficient ensuring stable frictional anisotropy. However, in real-world scenarios, the friction coefficient is influenced by the materials of the two objects in contact and is not easily controllable.

In summary, the analysis revealed the significant impacts of body height, bristle length, stiffness, reference angle, and friction coefficient on the frictional anisotropy of foxtail robot bristles. Notably, body height plays a critical role in determining buckling behavior and the resulting abrupt changes in frictional anisotropy. Shorter and stiffer bristles, smaller reference angles, and higher friction coefficients generally contribute to higher frictional anisotropy. However, beyond a certain threshold, higher frictional anisotropy does not necessarily translate to improved locomotion performance. Therefore, in the design of foxtail robots, once an adequate level of frictional anisotropy is achieved, the key considerations should focus on selecting appropriate reference angles and bristle lengths to ensure that the friction coefficient does not completely vanish at certain values. In addition, attention should be paid to the arrangement of bristles to prevent excessive weight on each bristle, thereby avoiding an excessively low body height.

### 3.2. Simulation of the Bristle Model

In this study, dynamic simulations were performed with the multi-link bristle model, as depicted in Figure 7. The equations of motion for the bristle system were derived using the Euler–Lagrange method mentioned in Section 2.3. Through these simulations, our objective was to enhance the comprehension of foxtail robot locomotion by analyzing bristle behavior.

#### 3.2.1. Simulation of Buckling Behavior during Backward Motion

**Figure 12:**
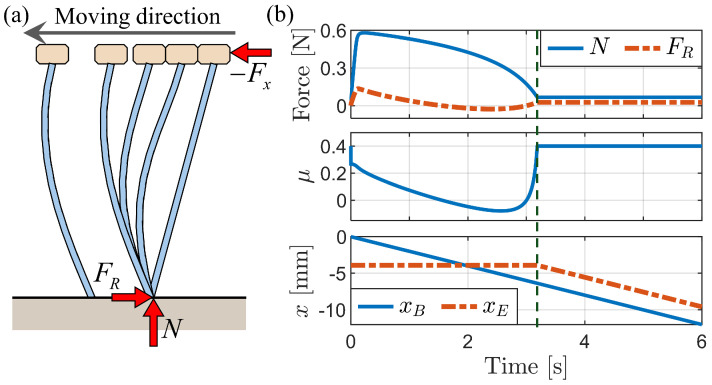
Simulation of backward motion at a constant velocity with $y_{B}$ = 14.5 mm. (**a**) Sequential images of bristle deformation at different time instances. (**b**) Simulation result graphs.

Figure 12a illustrates the bristle deformation process over time due to backward motion at a constant velocity of ${\dot{x}}_{B}=-2\;\mathrm{mm}/s$ while maintaining a fixed body height when $F_{y}=-0.001$ N at the start. The bristle end remains stationary until the complete buckling of the bristles occurs. Figure 12b depicts graphs of the normal force, friction force, friction coefficient, $x_{B}$, and $x_{E}$ over time. Notably, a substantial normal force was observed during the initial stages of deformation, preceding full buckling. During this simulation, the vertical external force ($F_{y}$) was calculated as necessary to maintain a fixed body height.

**Figure 13:**
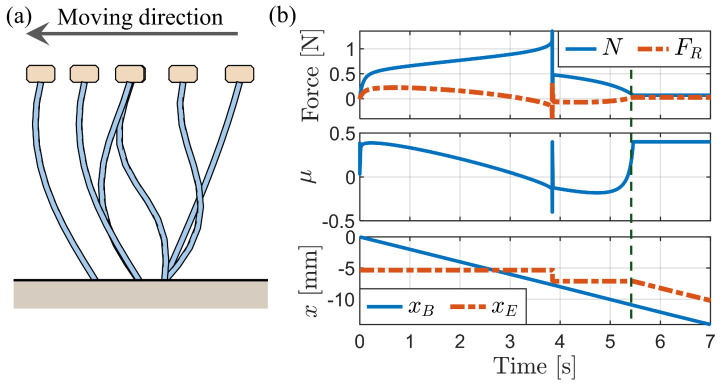
Simulation of backward motion at a fixed body height of 14 mm. (**a**) Sequential images of bristle deformation at different time instances. (**b**) Graphs showing changes in normal force, friction force, and friction coefficient.

Figure 13 shows the simulation results when the fixed body height is reduced to 14 mm. This reduction requires a larger vertical external force to maintain the height in contrast to the case of $F_{y}=-0.001$ N at a height of approximately 14.5 mm (Figure 12). The deformation starts from the middle of the bristles, forming an “S” shape. Subsequently, the bristles undergo a rapid transition from the “S” shape to a “C” shape, accompanied by a slight movement of the bristle end due to the release of elastic energy. After this transition to the “C” shape, no further movement of the bristle end is observed until complete buckling occurs.

**Figure 14:**
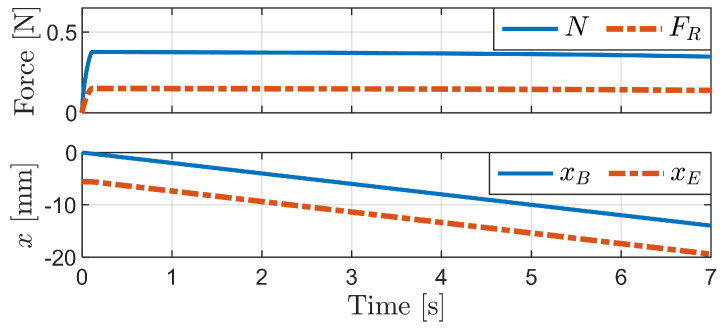
Simulation of backward motion at a fixed body height of 13.9 mm.

Figure 14 depicts the simulation results obtained for a fixed body height of 13.9 mm, which is below the critical height for buckling. The graph of bristle end movement ($x_{E}$) reveals that the bristles move backward without significant alterations in their shape, indicating the absence of buckling. In addition, the normal force remains relatively constant throughout the motion.

The simulations of bristle deformation during backward motion highlighted the critical role of buckling in the locomotion mechanism of foxtail robots. The results demonstrate that the bristle end remains stationary until complete buckling occurs, indicating that the robot’s actual movement is determined by the displacement of the bristle end rather than the displacement of the body. Furthermore, the simulations reveal a critical load required for buckling, below which the bristles exhibit negligible deformation. Furthermore, it was observed that the friction anisotropy in the buckling scenario surpasses the values calculated based on the stable state after buckling. These findings provide valuable insights into the complex interplay between bristle deformation, frictional anisotropy, and robot locomotion, emphasizing the importance of considering these factors in the design and control of foxtail robots.

#### 3.2.2. Press-and-Release Simulation

As mentioned in Section 1, the actual foxtail can be observed to move when the action of grasping and releasing by hand is repeated. However, interestingly, simulations involving models composed of either a single bristle or multiple identical bristles reveal a minimal translational motion of the body. This phenomenon persisted even when the action of pressing and releasing the bristles was repeated.

**Figure 15:**
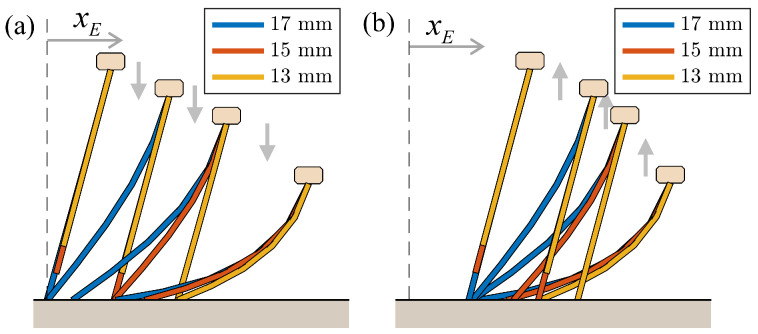
Simulation of the press-and-release action on a multi-bristle model with three different bristle lengths (13 mm, 15 mm, and 17 mm). Each bristle is composed of five links. (**a**) Bristle deformation during the pressing phase. (**b**) Bristle deformation during the release phase.

Conversely, when simulations were performed on a model comprising bristles of varying lengths, as shown in Figure 15, significant disparities occurred. The model consisted of three bristles with lengths of 13, 15, and 17 mm under a gradually increasing vertical load. As the load increased (Figure 15a), the longest bristle first made contact with the ground, followed sequentially by the medium and the shortest bristles. During this process, the body exhibited gradual forward movement. However, when the load was removed (Figure 15b), the model reverted to its original shape, aligning with the position of the longest bristle, with no further movement.

**Figure 16:**
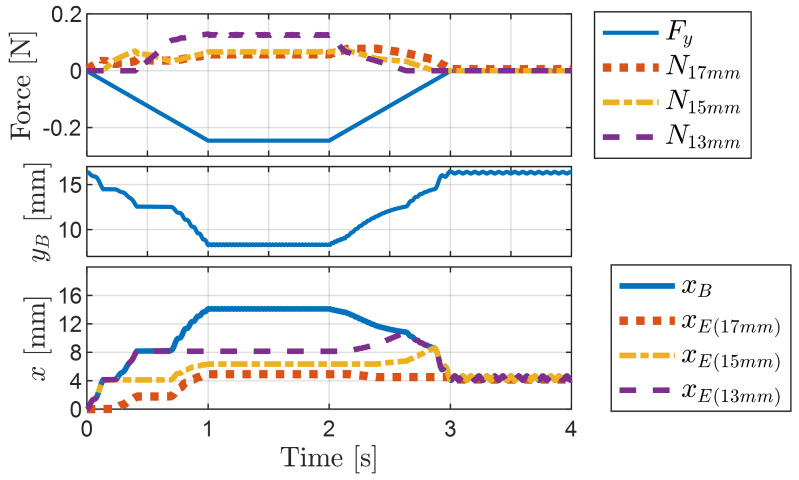
Graphs showing changes in the pressing force, normal forces, body height, and horizontal displacements of the body and the ends of each bristle over time during the press-and-release simulation. The initial values of the horizontal displacements were set to zero for comparison.

Figure 16 shows the graphs corresponding to the press-and-release simulation shown in Figure 15. These graphs illustrate variations in the pressing force (vertical external force), normal forces acting on bristles of various lengths, body height ($y_{B}$), and the horizontal displacements of the body ($x_{B}$) and the ends of each bristle ($x_{E(17mm)},x_{E(15mm)},x_{E(13mm)}$) over time. Throughout the progression of pressing and release actions, observable changes occurred in these variables.

During the pressing phase, the bristles previously in contact are pulled forward by the newly contacted bristle. This phenomenon is evident from the sequential increases in the normal forces acting on the bristles and the corresponding forward movements of the bristle ends. At each stage, the bristle with the highest normal force serves as a reference for the movement of the other bristles.

**Figure 17:**
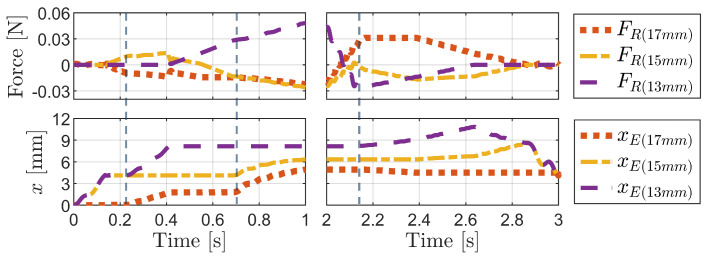
Graphs showing the changes in the friction forces and horizontal displacements of the ends of each bristle over time during the press-and-release simulation.

Figure 17 illustrates a comparison of the friction forces acting on each bristle and the movement of the bristle ends during two distinct phases: the pressing phase (0 to 1 s) and the release phase (2 to 3 s). While the normal force specifically identifies the bristle experiencing the force, the friction force provides insights into the dynamic behavior of the bristles as they move. A negative friction force indicates that the corresponding bristle is either forwarding or resisting a forward-directed force, whereas a positive friction force indicates that the bristle is either moving backward or opposing a backward-directed force. In particular, for tilted bristles, the frictional anisotropy discussed in Section 3.1 implies that a greater force is required for backward motion. Therefore, it can be inferred that bristles experiencing a positive friction force primarily resist the backward motion rather than actually move backward.

Figure 17 illustrates how the friction force graphs predict bristle movement based on the sign of the friction force and the interaction between bristles. During the pressing phase, the 15 mm bristle experiences a positive friction force, resisting backward motion and allowing the forward advancement of the 17 mm bristle, which experiences a negative friction force. As the 13 mm bristle makes contact, it assumes the responsibility of resisting backward motion, thereby enabling the forward movement of the 15 mm and 17 mm bristles. During the release phase, the friction force on the 17 mm bristle becomes positive, serving as a reference for the other bristles to advance forward. This approach can be extended to the analysis of foxtail robot simulations in Section 4, which provides insights into the locomotion mechanism of the robot.

**Figure 18:**
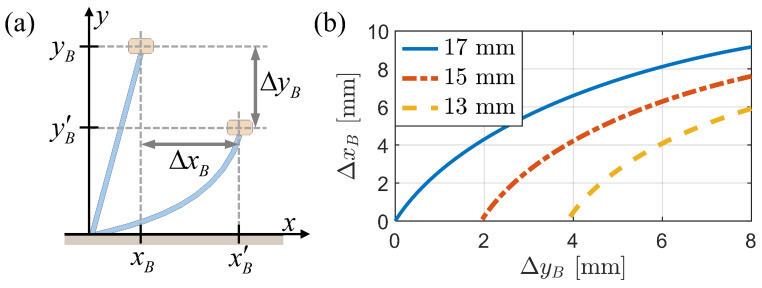
Relationship between pressing depth and body displacement for different bristle lengths. (**a**) Illustration of bristle deformation and the corresponding horizontal displacement of the body ($\Delta x_{B}$) for different pressing depths ($\Delta y_{B}$). (**b**) Graph showing $\Delta x_{B}$ as $\Delta y_{B}$ for bristles with lengths of 13, 15, and 17 mm.

Figure 18 illustrates the nonlinear relationship between the change in body height ($\Delta y_{B}$) and the horizontal displacement of the body ($\Delta x_{B}$) across various bristle lengths. The slope of the curve varies with the change in height and varies with bristle length. Simultaneous changes in the heights of bristles of different lengths, along with frictional anisotropy, contribute to the forward motion of the robot.

**Figure 19:**
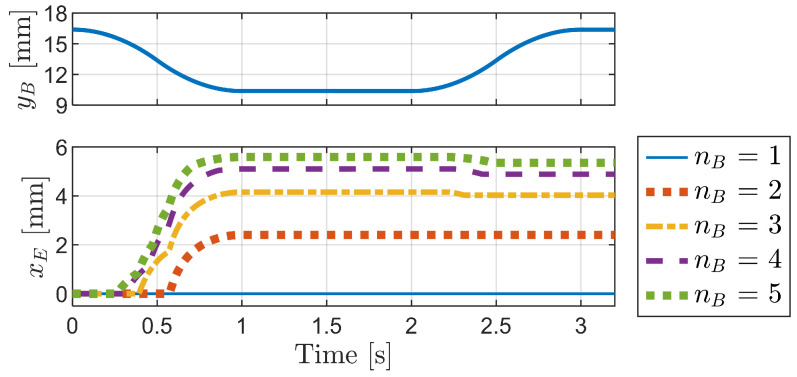
Comparison of the pressing depth and forward travel distance of the longest bristle’s end ($x_{E}$) for models with different numbers of bristles ($n_{B}$) from 1 to 5.

Figure 19 compares the pressing depth and forward travel distance of the longest bristle end ($x_{E}$) across models with varying numbers of bristles ($n_{B}$), ranging from 1 to 5. The findings indicate that the single-bristle model exhibits no forward motion when pressed and released, whereas the forward travel distance increases as the number of bristles increases from two to five.

The simulation results indicate that the diversity of bristle lengths and angles, specifically the nonuniformity of the bristle array, significantly influences the locomotion mechanism of foxtail robots. This nonuniformity contributes to the generation of propulsive force in foxtail robots, highlighting the importance of prioritizing the nonuniformity of the bristle array during design considerations.

## 4. Foxtail Robot Model

**Figure 20:**
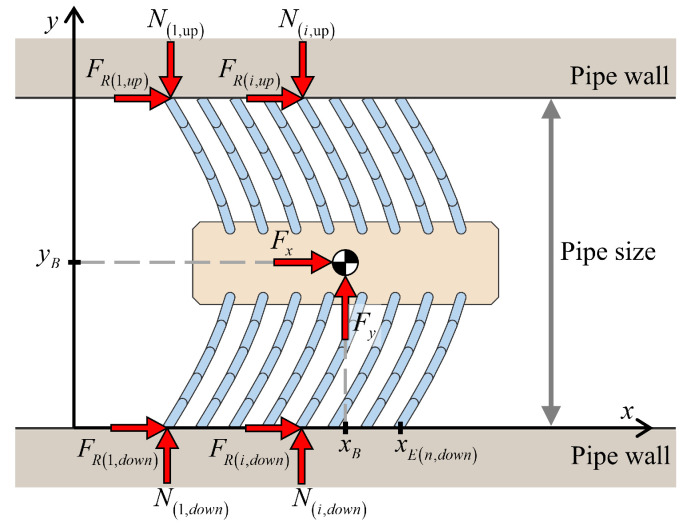
Schematic of the basic foxtail robot model inside a pipe.

In this study, dynamic simulations were performed on an entire foxtail robot system that incorporates multiple bristles, as shown in Figure 20. The robot was assumed to be positioned within a pipe, with the upper and lower bristle tips in contact with the pipe walls. Each bristle comprised five links, and the robot consisted of 16 bristles, with eight bristles each on the upper and lower sides. Simulations were conducted for the six cases classified in Section 1.

### 4.1. Passive Locomotion by External Forces

This section explores the impact of bristle friction anisotropy on the propulsion mechanism of passively actuated foxtail robots. We present simulation results for robots driven by externally applied forces and analyze their dynamic response characteristics under various load conditions. The robot body was set to have dimensions of 31.5 mm in width, 8.5 mm in height, and 2 mm in thickness and was assumed to be made of PLA. The weight and moment of inertia of the robot were calculated based on these specifications. In all cases, except for the pipe height change scenario, the pipe height was set to 34 mm.

#### 4.1.1. Horizontal External Force

**Figure 21:**
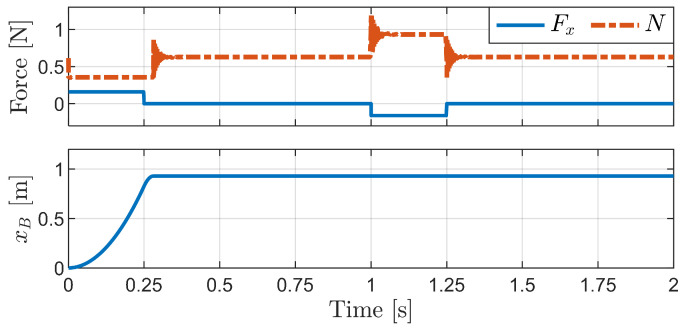
Simulation results for the horizontal external force case.

Figure 21 shows the simulation results for the horizontal external force case. The first graph represents the applied horizontal external force ($F_{x}$) and the sum of the normal force of all bristles (*N*) over time. The second graph illustrates the horizontal displacement of the robot body ($x_{B}$), which indicates the overall movement of the robot. In the simulation of the foxtail robot, the robot transitions from Phase 1 to Phase 2 and then returns to its original shape in Phase 1. This process is observable through the movement of the robot body ($x_{B}$).

The results indicate that when a forward force is applied, the robot experiences rapid forward movement. During this period, the total normal force decreases. However, when the same force is applied in the backward direction, the robot remains stationary, and the total normal force is increased. This phenomenon highlights the significance of frictional anisotropy, which enables the robot to move exclusively in the forward direction under horizontal external forces.

#### 4.1.2. Vertical External Force

**Figure 22:**
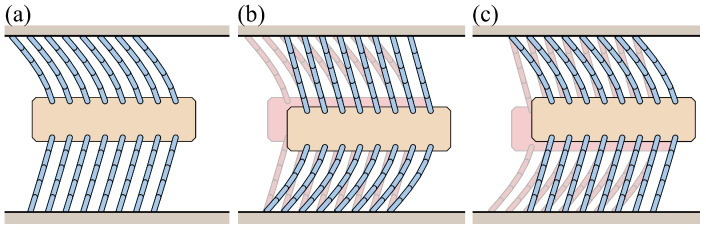
Illustration of foxtail robot deformation under vertical external force: (**a**) Phase 1, (**b**) Phase 1 to Phase 2, (**c**) Phase 2 to Phase 1.

Figure 22 illustrates the deformation of the foxtail robot under vertical external forces. In Phase 1, an upward force compresses the upper bristles, and this compressed shape persists even after the force is removed. In Phase 2, a downward force is applied, causing the previously compressed upper bristles to release and move forward. When an upward force is applied again, the robot transitions from Phase 2 to Phase 1, and the lower bristles move forward during this transition.

**Figure 23:**
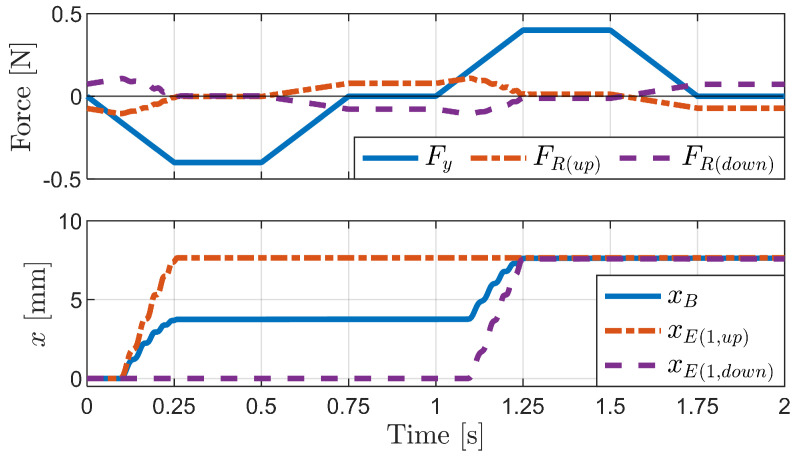
Simulation results for the vertical external force case.

Figure 23 shows the simulation results for the vertical external force case. The first graph depicts the applied vertical external force ($F_{y}$) and the sum of the friction forces of the upper ($F_{R(up)}$) and lower bristles ($F_{R(down)}$) over time. This visualization illustrates how the friction forces on the upper and lower bristles change in response to the applied vertical force. The second graph shows the horizontal displacement of the robot’s body and the first upper and lower bristle ends ($x_{B}$, $x_{E(1,up)}$, $x_{E(1,down)}$). This visualization illustrates the forward motion of the robot during the application of the vertical force.

The force was gradually applied, maintained at the peak value, and then removed. This force profile induces changes in the friction forces acting on the upper and lower bristles. When a sufficient downward force is applied, the upper bristles are released, resulting in negative friction and forward motion, while the lower bristles experience positive friction as they resist downward motion. Conversely, when an upward force is applied, the upper bristles experience positive friction as they resist the upward motion, while the lower bristles experience negative friction and move forward. As discussed in Section 3.2.2, the bristles with positive friction resist the motion, allowing the bristles with negative friction to advance. This alternating resistance and forward motion of the upper and lower bristles translates into a step-wise forward motion of the robot body, as evident from the horizontal displacement graph.

#### 4.1.3. Change in Pipe Height

In the case of a foxtail robot with uniform bristle lengths, the robot does not move even when the pipe compresses and releases the robot. This is because all bristles experience the same amount of compression and deformation, resulting in no net propulsive force.

**Figure 24:**
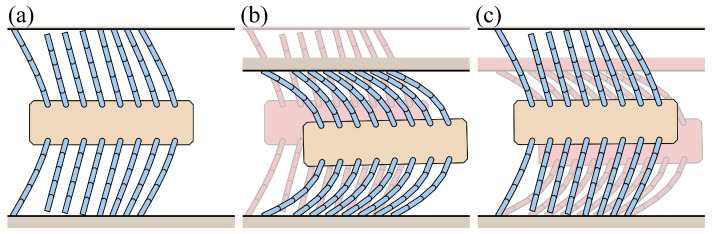
Illustration of the foxtail robot with varying bristle lengths under pipe height change: (**a**) Phase 1, (**b**) Phase 1 to Phase 2, (**c**) Phase 2 to Phase 1.

Figure 24 illustrates the case of pipe height change with varying bristle lengths. The bristles have the same reference angle, with the first and eighth bristles having a length of 17 mm, the second bristle having a length of 13 mm, and the bristle lengths increasing in an arithmetic sequence from the second to the eighth bristle. With decreasing pipe height, the longest bristle establishes initial contact with the pipe wall, followed by the sequential engagement of the remaining bristles. As each bristle makes contact, its tip advances forward. After reaching its target low height, the pipe height stops descending. During the subsequent return to its original height, the shorter bristles detach from the pipe wall. However, the positions of the bristle ends remain unchanged.

**Figure 25:**
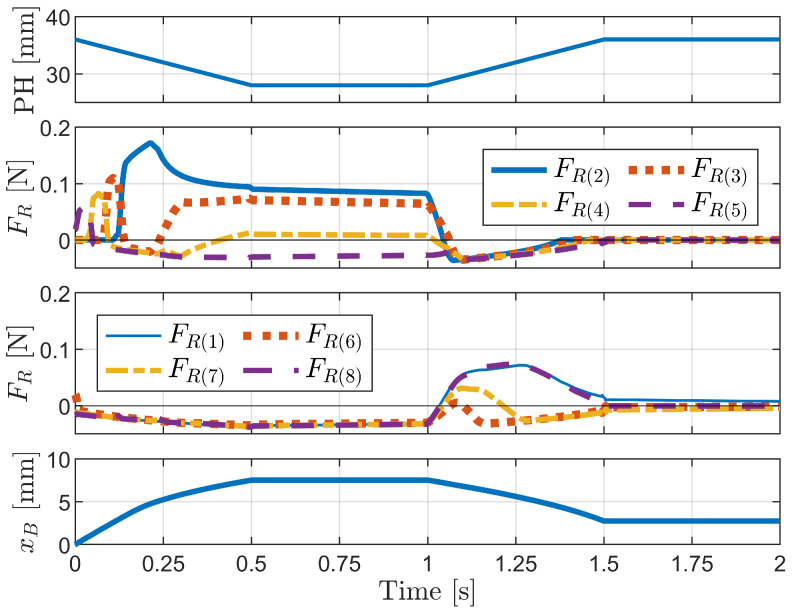
Simulation results for the pipe height (PH) change case.

Figure 25 shows the pipe height (PH) and the friction forces for the second, third, fourth, and fifth bristles (shorter bristles) and the first, sixth, seventh, and eighth bristles (longer bristles). The friction forces represent the sum of the values for the upper and lower bristles of each index. The last graph illustrates the horizontal displacement of the body ($x_{B}$).

The pipe height change case demonstrates the role of bristle diversity in achieving effective propulsion, similar to the press-and-release scenario discussed in Section 3.2.2. As the pipe height decreases, the shorter bristles (second to fifth) sequentially come into contact with the pipe wall, exhibiting large positive friction forces and serving as a reference for the other bristles to advance forward. The longer bristles (first, sixth, seventh, and eighth) remain in contact with the pipe wall throughout the process, resulting in an increase in friction forces. When the pipe height returns to its original state, the friction forces of the shorter bristles decrease to negative values, while those of the longer bristles increase and become positive, indicating their role as a reference for the robot to return to its original state. The sequential compression and release of bristles of varying lengths result in the forward motion of the robot, highlighting the importance of bristle diversity, friction anisotropy, and dynamic response in the locomotion mechanism of foxtail robots.

The simulation results for the passive locomotion cases underscore the importance of bristle friction anisotropy and the dynamic response of the robot under various external force conditions. Specifically, the horizontal external force case highlights the unidirectional motion of the robot due to frictional anisotropy. Conversely, the vertical external force and pipe height change cases demonstrate the forward motion of the robot through the sequential compression and release of the bristles. These findings provide valuable insights into the locomotion mechanism of foxtail robots and the role of bristle diversity in achieving effective propulsion.

### 4.2. Active Locomotion by Internal Actuation

This section presents simulation results for cases in which the foxtail robot actively moves using internal actuation mechanisms. The dynamic response of the robot and the role of bristle diversity in generating propulsive force were analyzed for each case.

#### 4.2.1. Inchworm Mechanism

**Figure 26:**
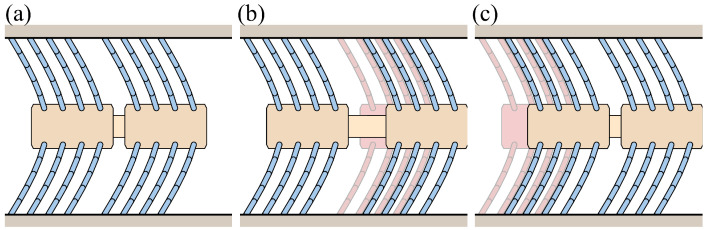
Illustration of the foxtail robot with the inchworm mechanism: (**a**) Phase 1, (**b**) Phase 1 to Phase 2, (**c**) Phase 2 to Phase 1.

The inchworm mechanism is a combination of the inchworm robot design and the foxtail mechanism, which has been previously studied in foxtail robots [8]. Figure 26 illustrates the foxtail robot with the inchworm mechanism. The robot’s body is divided into front and rear segments, each with four pairs of upper and lower bristles. A linear link connects these segments, allowing for an adjustable distance between them. Each body segment had dimensions of 14 mm in width, 8.5 mm in height, and 2 mm in thickness, and the weight and inertia were calculated accordingly.

**Figure 27:**
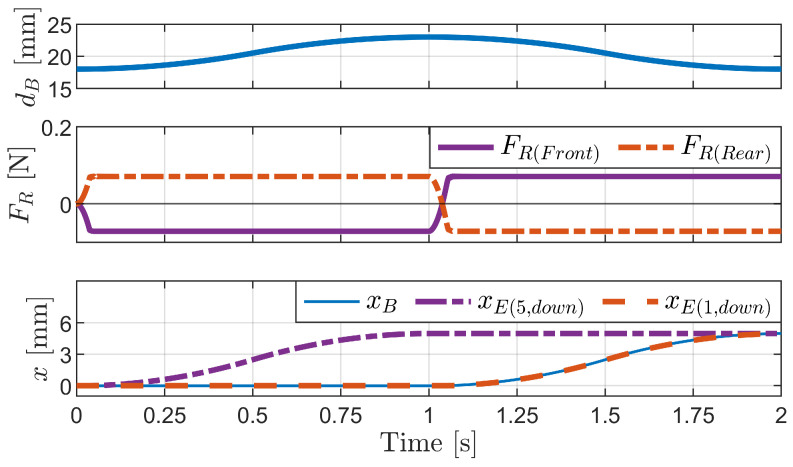
Simulation results for the inchworm mechanism.

Figure 27 shows the simulation results for the inchworm mechanism. The first graph illustrates the distance between the body segments ($d_{B}$) over time. The second and third graphs display the sum of the friction forces of the front and rear bristles, respectively. The fourth graph illustrates the horizontal displacement of the front and rear bristle ends, where $x_{E(1,down)}$ represents the rear bristles, and $x_{E(5,down)}$ represents the front bristles. The horizontal displacement of the robot body ($x_{B}$) is also shown, which is based on the rear body segment and thus exhibits the same change as the rear bristles.

As the distance between the body segments increases, the friction force on the rear bristles increases and becomes positive, whereas the friction force on the front bristles decreases and becomes negative. Consequently, the front bristles advance forward, while the rear bristles remain stationary. Conversely, as the distance between the body segments decreases, the friction force on the front bristles increases and becomes positive, while the friction force on the rear bristles decreases and becomes negative. Consequently, the rear bristles move forward while the front bristles remain stationary.

These results can be attributed to the anisotropy of bristle friction. Due to the frictional anisotropy, when comparable magnitudes of friction forces are applied to the bristles, the positive friction force resists backward motion, while the negative friction force is insufficient to prevent forward motion. Consequently, the bristles experiencing negative friction forces advance forward, while those experiencing positive friction forces remain stationary or resist backward motion.

The inchworm mechanism effectively leverages the frictional anisotropy of the bristles to generate a net propulsive force. By alternating the compression and extension of its body segments, the robot coordinates the movement of its front and rear bristles, achieving forward locomotion.

#### 4.2.2. Phase-Difference Mechanism

**Figure 28:**
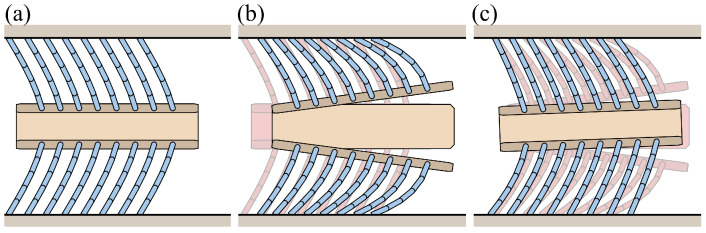
Illustration of the foxtail robot with the phase-difference mechanism: (**a**) Phase 1, (**b**) Phase 1 to Phase 2, (**c**) Phase 2 to Phase 1.

**Figure 29:**
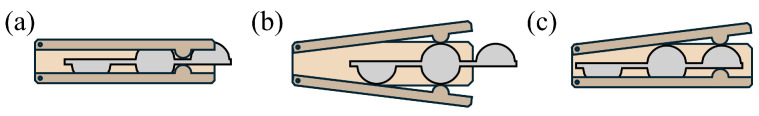
Schematic of the phase-difference mechanism: (**a**) basic structure, (**b**) simultaneous movement of upper and lower sub-bodies, (**c**) movement of only the upper side of the sub-bodies.

The phase-difference mechanism has been studied for locomotion in foxtail robots [10]. Figure 28 illustrates the phase changes in the robot operating with the phase-difference mechanism. In this mechanism, sub-bodies are connected to the main body through the joints. A central bar determines the phase by being pushed or pulled by a linear motor, as shown in Figure 29. The main body has dimensions of 36 mm in width, 8.5 mm in height, and 2 mm in thickness, whereas the sub-bodies have dimensions of 36 mm in width, 1 mm in height, and 2 mm in thickness. The weight and inertia were calculated accordingly.

**Figure 30:**
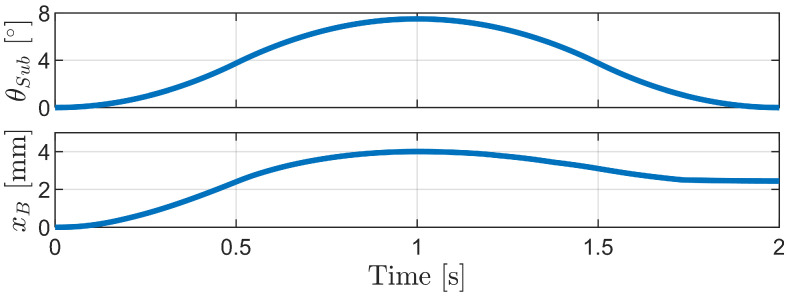
Simulation results for the phase-difference mechanism: the angle of the sub-body ($\theta_{Sub}$) over time and the horizontal displacement of the robot body ($x_{B}$) over time.

**Figure 31:**
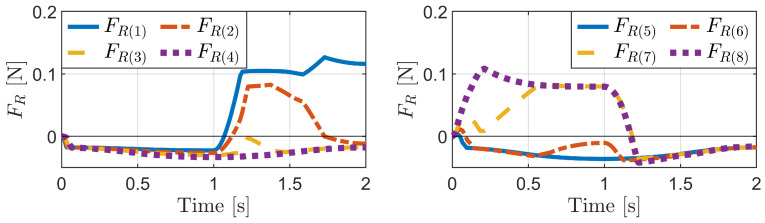
Simulation results for the phase difference mechanism: friction forces of the bristles.

Figure 30 and Figure 31 show the simulation results for the phase-difference mechanism. The robot advances from 0 to 1 s as the sub-bodies spread apart and then slightly retreats from 1 to 2 s as they return to their original state. This advancement can be attributed to the variation in compression among the bristles.

The movement of the robot with the phase-difference mechanism is similar to that observed in Section 4.1.3, where the pipe height changes. Although the mechanism in this case uses body expansion instead of pipe height changes, the underlying principle remains identical. However, real-world applications may have limitations in dynamically adjusting pipe height, making the phase-difference mechanism a more adaptable solution.

In Figure 31, the friction forces of the seventh and eighth bristles exhibit a positive increase during the transition from Phase 1 (0 s) to Phase 2 (1 s). These frictional forces decrease to negative values thereafter. Subsequently, the friction forces of the first and second bristles increase and become positive from 1 s onward. The other bristles exhibit negative friction forces. This indicates that from 0 to 1 s, the seventh and eighth bristles serve as the reference, allowing the other bristles to advance forward. From 1 s (Phase 2) to 2 s (Phase 1), the first and second bristles become the reference, enabling the other bristles to return to their original state.

During the transition from Phase 1 to Phase 2, the seventh and eighth bristles experience the most compression and undergo the largest change, playing a role similar to the short bristles described in Section 3.2.2. Conversely, the first and second bristles are the least compressed and experience minimal change, serving as a reference during the return phase.

The phase difference mechanism effectively leverages the diversity in bristle compression to generate a propulsive force. The bristles experiencing positive friction forces resist backward motion, whereas those with negative friction forces advance forward. By alternately compressing and extending the sub-bodies, the robot achieves forward locomotion through coordinated bristle movement.

#### 4.2.3. Carangiform Locomotion Mechanism

**Figure 32:**
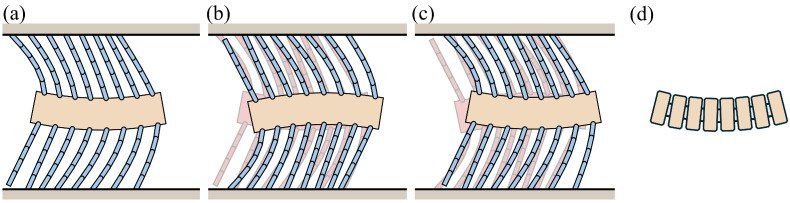
Illustration of the foxtail robot with the carangiform locomotion mechanism: (**a**) Phase 1, (**b**) Phase 1 to Phase 2, (**c**) Phase 2 to Phase 1. (**d**) Schematic of the carangiform locomotion mechanism.

The carangiform locomotion mechanism, inspired by the swimming motion of fish, has been explored in foxtail robots [1]. Figure 32 illustrates the phase changes in a robot operating with the carangiform locomotion mechanism, which mimics this motion by bending the body [1]. The robot body is divided into multiple segments connected by joints, allowing for a smooth bending motion, as shown in Figure 32d. In the simulations, there were eight body segments, one for each pair of upper and lower bristles. Each body segment had dimensions of 3.6 mm in width, 8.5 mm in height, and 2 mm in thickness, and the weight and inertia were calculated accordingly.

**Figure 33:**
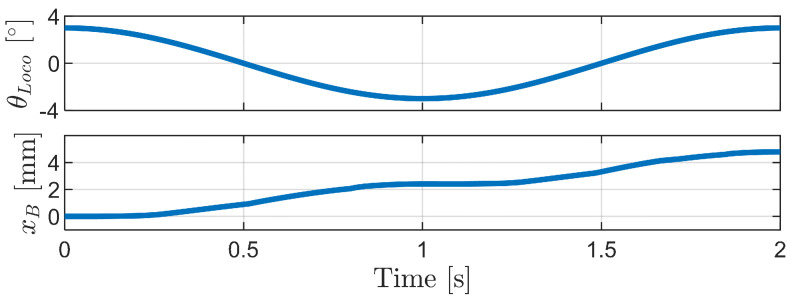
Simulation results of the carangiform locomotion mechanism: bending angle between body segments ($\theta_{Loco}$) and horizontal displacement of the robot body ($x_{B}$) over time.

**Figure 34:**
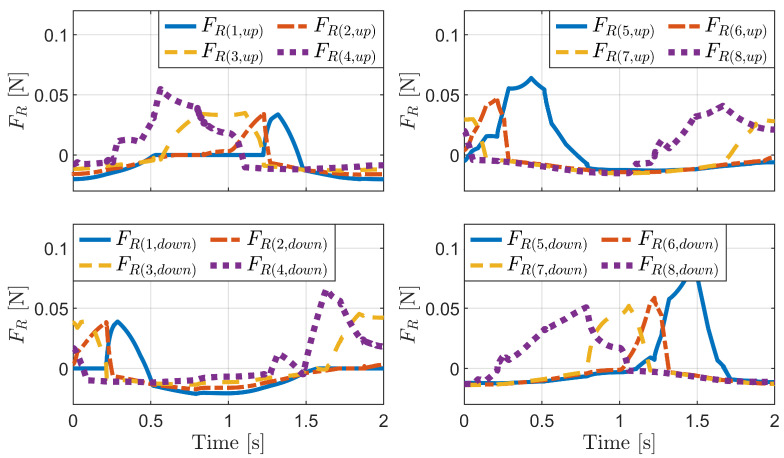
Simulation results of the carangiform locomotion mechanism: friction forces of the bristles.

Figure 33 and Figure 34 present the simulation results for the carangiform locomotion mechanism. As the robot bends in opposite directions, it continuously advances forward. This forward motion can be attributed to the diversity in bristle compression and angle changes caused by the varying angles of each body segment during the bending motion. This diversity leads to differences in frictional forces among the bristles, resulting in a net propulsive force.

In the carangiform locomotion mechanism, the reference angle of the bristles and the height (i.e., $y_{B}$ in Section 3.2.2) vary for all 16 bristles. This diversity in changes facilitates the robot’s continuous forward motion throughout the period. The friction forces of the bristles located at the ends (first, second, seventh, and eight) and in the central region (third, fourth, fifth, and sixth) of the robot alternately increase and decrease during the phase transitions, as shown in Figure 34. The sequential pressing and releasing of the bristles in different regions of the robot body result in the coordinated forward movement of the robot.

The carangiform locomotion mechanism effectively leverages the diversity in bristle compression and angle changes to generate a net propulsive force. This underscores the critical role of bristle diversity in the locomotion of foxtail robots and the potential of bioinspired designs for achieving efficient and adaptive robotic locomotion.

The simulation results for the active locomotion scenarios underscore the effectiveness of internal actuation mechanisms in generating propulsive force, thereby enabling the forward movement of the robot. The inchworm mechanism relies primarily on the anisotropic friction properties of the bristles to achieve locomotion. Conversely, the phase-difference and carangiform locomotion mechanisms leverage both anisotropic friction properties and diversity in bristle compression and angle changes to generate propulsive force. These findings highlight the importance of using the unique properties of bristles and the role of internal actuation in the design and control of foxtail robots for efficient and adaptable locomotion across diverse environments.

## 5. Discussion

The findings of this study shed light on the locomotion principles of foxtail robots and provide practical insights for their design and control. The role of bristle diversity in generating propulsive force through nonuniform ground contact and frictional anisotropy is a key takeaway that can guide future developments in the field of biomimetic robotics, particularly in the design of robots inspired by the locomotion of biological organisms.

Incorporating bristle diversity into the design of foxtail robots can lead to enhanced locomotion performance and adaptability to various environments. This can be achieved by intentionally combining bristles with different characteristics during the design process.

The generalized mechanism of propulsive force generation through bristle diversity, as elucidated by the simulations, offers a framework for understanding and optimizing the locomotion of foxtail robots. This mechanism can be leveraged to design foxtail propulsion systems for a wide range of robotic applications, from pipe inspection to search and rescue operations in cluttered environments. Previous research has already demonstrated the adaptability of foxtail robots enabling their operation in diverse environments [1].

Similar to the previous integration of inchworm robots and foxtail mechanisms, as discussed in Section 4.2.1, integrating foxtail mechanisms into existing biomimetic robot designs, such as snake robots, presents a promising approach. For instance, integrating snake robots could provide supplementary benefits like improved propulsion and adaptability to diverse terrains. Notably, this integration would not necessitate additional power sources, as the foxtail mechanism can operate by exploiting the existing motions of the host robot. In incorporating foxtail bristles, biomimetic robots could achieve enhanced mobility and efficiency without significant modifications to their core design.

For robots that only require forward motion, the addition of foxtail bristles can provide significant benefits without the need for complex modifications. In such cases, the foxtail mechanism can enhance the robot’s locomotion capabilities.

However, the unidirectional nature of the foxtail mechanism may pose a limitation for some applications. In applications where bidirectional motion is necessary, alternative solutions need to be explored to address the limitations of the foxtail mechanism due to its frictional anisotropy, which hinders backward motion. As alternatives, the bristles are retracted or the reference angle is changed in the opposite direction during backward motion. Incorporating such mechanisms would require additional actuation systems. Therefore, when integrating foxtail mechanisms into existing robots, researchers and designers must carefully assess the specific requirements of the robot and weigh the potential benefits against the added complexity of the system. Further investigation into optimizing the integration of foxtail mechanisms will be crucial for expanding the capabilities and applications of foxtail robots.

**Figure 35:**
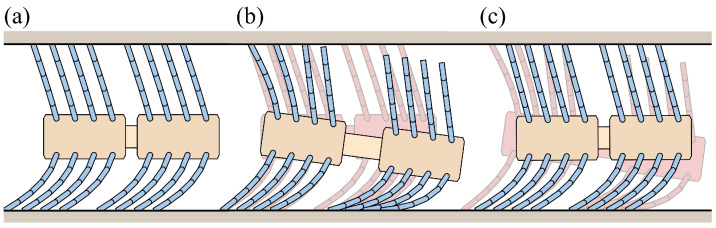
Illustration of foxtail robot with the inchworm mechanism under 80-fold increased body weight: (**a**) Phase 1, (**b**) Phase 1 to Phase 2, (**c**) Phase 2 to Phase 1.

If additional components such as motors are incorporated into the robot, the body weight will further increase. To briefly examine the impact of such weight increase, we conducted simulations for the inchworm mechanism, assuming an extreme case where the body weight was increased 80-fold, and the pipe height was lowered to 32 mm. The results show that during the transition from Phase 1 to Phase 2, i.e., when the body is extending, the increased weight caused the robot’s body to tilt forward, as shown in Figure 35b.

**Figure 36:**
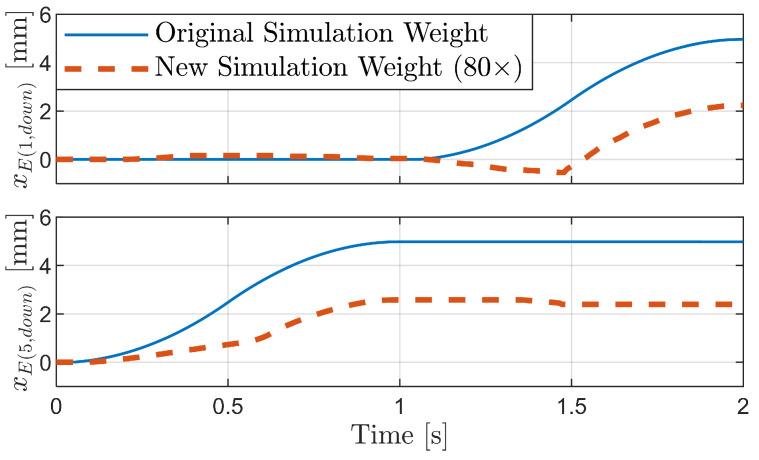
Simulation results for the inchworm mechanism with original and 80-fold-increased body weight.

This tilting limited the forward motion of the front legs during the extension process, resulting in the robot advancing only about half the distance compared to the original case, as can be seen in Figure 36. This suggests that the weight increase induced bristle deformation, constraining the generation of propulsive force. However, it was observed that the locomotion principle of moving forward through frictional anisotropy was still maintained even with the increased weight. For a more accurate analysis, systematic simulations and experiments under various weight conditions would be necessary. Through such additional studies, strategies for optimizing the weight factor in the design and control of foxtail robots could be explored.

Despite these challenges, the current study established a solid foundation for understanding and exploiting the unique locomotion principles of foxtail robots. The insights gained from this work can drive further advancements in soft robotics and biomimetic engineering, ultimately leading to the development of more versatile, efficient, and adaptable robotic systems for real-world applications.

## 6. Conclusions

In this study, we conducted dynamic analyses and simulations to deepen our understanding of the locomotion mechanism of foxtail robots. Our research focused on two key aspects: elucidating the principle of frictional anisotropy in tilted bristles under the same friction coefficient and investigating the propulsion strategy of foxtail robots using bristle diversity.

Through the analysis of frictional anisotropy based on changes in major design parameters, we identified the critical influence of body height, bristle length, stiffness, reference angle, and friction coefficient on the frictional anisotropy of foxtail robot bristles. This interaction between contact nonuniformity and frictional anisotropy emerged as the core principle enabling the locomotion of foxtail robots.

Simulations of the bristle model revealed the crucial role of bristle diversity and nonuniformity in generating propulsive force. The press and release simulations demonstrated that variations in bristle length and angle contribute to the robot’s forward motion. These findings highlight the importance of designing robots with inherent bristle nonuniformity.

Dynamic simulations of the entire foxtail robot system under various scenarios further confirmed that bristle diversity is a key factor in the locomotion principles of the robot. The phase-difference and carangiform locomotion mechanisms demonstrated how variations in bristle compression and angle can be leveraged to generate propulsive force.

The insights gained from this study advance our understanding of foxtail robot locomotion principles and provide valuable guidelines for their design and control. The proposed simulation framework enables the quantitative prediction and optimization of foxtail robot performance, facilitating the development of more efficient and adaptable robotic systems.

In conclusion, this study provides a comprehensive analysis of foxtail robots, focusing on the roles of frictional anisotropy and bristle diversity in their locomotion, design considerations, and control strategies. The findings offer valuable insights for developing efficient and adaptable foxtail robots capable of navigating various challenging environments. The proposed approach and insights can be extended to the design and control of other soft robotic systems, paving the way for the development of versatile and adaptable robots.

## Figures and Tables

**Table 1:** Specifications.

Parameter	Value	Units
Material	PLA	
Bristle length *L*	15	mm
Bristle reference angle $\theta_{Ref}$	15	degree (°)
Bristle thickness *h*	0.4	mm
Bristle width *b*	0.4	mm
PLA density	1240	kg/m^3^
PLA damping ratio ζ	3	%
Kinetic friction coefficient $\mu_{k}$	0.4	
Characteristic velocity γ	0.001	mm/s
Area moment of inertia *I*	2.13 × 10^−3^	mm^4^
Young’s modulus of PLA *E*	3500	MPa
Ground stiffness $K_{G}$	10^4^	N/m
Ground damping $B_{G}$	75	N·s/m

**Figure A1:**
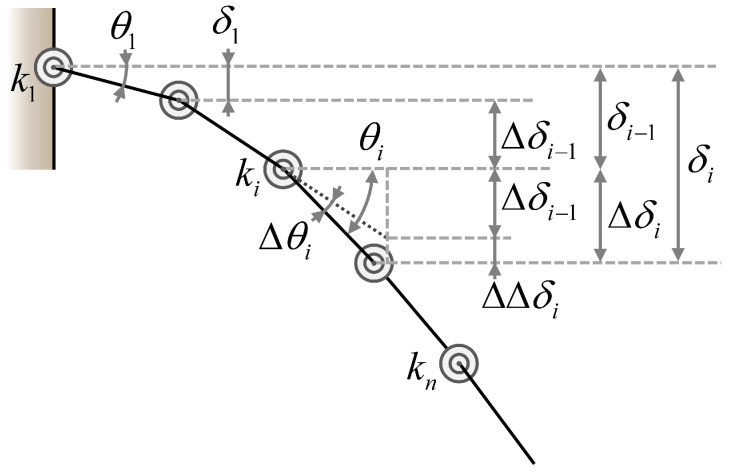
Schematic of the linkage-spring system, showing the absolute and relative rotation angles ($\theta_{1}$, $\Delta \theta_{1}$, $\Delta \theta_{i}$) and the stiffness of the *i*-th torsional spring ($k_{i}$).

## Data Availability

The data supporting the findings of this study are available within the article. Additional data related to the simulation results and modeling parameters used in this study are available from the corresponding author upon reasonable request.

## Abbreviations

The following abbreviations are used in this manuscript:

PLA	Polylactic Acid;
CAD	Computer-Aided Design;
FR	Force Ratio;
PH	Pipe Height.

## Appendix A. Derivation of Spring Stiffness for Linkage-Spring Model

This appendix presents a detailed derivation of the spring stiffness values for the linkage-spring model used to represent the bristles in the foxtail robot. Our goal was to calculate the stiffness of the torsional springs such that the model’s deformation closely resembles the deflection of a cantilever beam under the same loading conditions.

The deflection of a cantilever beam due to shear force is calculated according to the beam length *L*, Young’s modulus *E*, and area moment of inertia *I*. The bristle is modeled as a linkage-spring system consisting of *n* links and torsional springs between the links. The torque $M_{i}$ of the *i*-th torsional spring can be calculated by multiplying the relative rotation angle $\Delta \theta_{i}$ of the adjacent links by the stiffness $k_{i}$. As shown in Figure A1, for the first link, the absolute rotation angle $\theta_{1}$ becomes the relative rotation angle $\Delta \theta_{1}$, and the corresponding deflection $\delta_{1}$ can be approximated as the product of the link length $l_{L}=L/n$ and the rotation angle, as shown in Equation (A1). In addition, the moment $M_{1}$ acting on the first torsional spring is PL according to the cantilever beam theory, and through this, the stiffness $k_{1}$ of the first spring can be calculated as shown in Equation (A2).

(A1) $$\delta_{1}=\frac{P\left(3n-1\right)L^{3}}{6n^{3}EI}\approx \frac{L}{n}\theta_{1}$$

(A2) $$k_{1}\theta_{1}=M_{1}\Rightarrow k_{1}\frac{n}{L}\delta_{1}=PL\Rightarrow k_{1}=\frac{6n^{2}EI}{\left(3n-1\right)L}$$

For the links after the second link, the relative rotation angle between adjacent links is the difference between the absolute rotation angles of the two links. As shown in Figure A1, the deflection change $\Delta \delta_{i}$ of the *i*-th link is expressed by Equation (A3), and the change in the deflection change $\Delta \Delta \delta_{i}$ is expressed by Equation (A4). In this context, $\Delta \delta_{1}=\delta_{1}$. When the rotation angle is small, it can be approximated as sinθ≈θ, so the relationship between the relative rotation angle and ΔΔδ is as shown in Equation (A5). Meanwhile, the moment $M_{i}$ acting on the *i*-th spring is given by Equation (A6) according to the cantilever beam theory, and this will be equal to the torque of the *i*-th spring. Therefore, the stiffness $k_{i}$ of the *i*-th spring is derived as shown in Equation (A7), which does not include *i*. In other words, it can be seen that the stiffness $k_{Else}$ of all springs except the first one is the same.

(A3) $$\Delta \delta_{i}=\delta_{i}-\delta_{i-1}=\frac{PL^{3}}{6EIn^{3}}\left\{\left(6i-3\right)n-\left(3i^{2}-3i+1\right)\right\}\;(\mathrm{for}\;i\geq 2)$$

(A4) $$\Delta \Delta \delta_{i}=\Delta \delta_{i}-\Delta \delta_{i-1}=\frac{PL^{3}\left(n-i+1\right)}{EIn^{3}}\;(\mathrm{for}\;i\geq 2)$$

(A5) $$\Delta \Delta \delta_{i}\approx \frac{L}{n}\Delta \theta_{i}$$

(A6) $$M_{i}=\frac{n-i+1}{n}PL$$

(A7) $$k_{i}\Delta \theta =M_{i}\Rightarrow k_{i}\frac{n}{L}\Delta \Delta \delta_{i}=\frac{n-i+1}{n}PL\Rightarrow k_{i}=\frac{nEI}{L}\;(\mathrm{for}\;i\geq 2)$$

In applying the derived stiffness values to the torsional springs in the linkage-spring model, the deformation of the model under applied forces closely resembles the deflection of a cantilever beam. This approach enables the efficient simulation of large bristle deformations and their interactions with the ground in the foxtail robot model.
